# Co-overexpression of geraniol-10-hydroxylase and strictosidine synthase improves anti-cancer drug camptothecin accumulation in *Ophiorrhiza pumila*

**DOI:** 10.1038/srep08227

**Published:** 2015-02-04

**Authors:** Lijie Cui, Xiaoling Ni, Qian Ji, Xiaojuan Teng, Yanru Yang, Chao Wu, David Zekria, Dasheng Zhang, Guoyin Kai

**Affiliations:** 1 Laboratory of Plant Biotechnology, Development Center of Plant Germplasm Resources, College of Life and Environment Sciences, Shanghai Normal University, Shanghai 200234, China; 2 Department of General Surgery, Zhongshan Hospital, Shanghai Medical College, Fudan University, Shanghai 200032, China; 3 Shanghai Chenshan Plant Science Research Center, Shanghai Chenshan Botanical Garden, Chinese Academy of Sciences, Shanghai201602, China; 4 Zhejiang Provincial Key Laboratory for Genetic Improvement and Quality Control of Medicinal Plants, Hangzhou Normal University, Hangzhou 310018, China

## Abstract

Camptothecin (CPT) belongs to a group of monoterpenoidindole alkaloids (TIAs) and its derivatives such as irinothecan and topothecan have been widely used worldwide for the treatment of cancer, giving rise to rapidly increasing market demands. Genes from *Catharanthus roseus* encoding strictosidine synthase (STR) and geraniol 10-hydroxylase (G10H), were separately and simultaneously introduced into *Ophiorrhiza pumila* hairy roots. Overexpression of individual *G10H* (G lines) significantly improved CPT production with respect to non-transgenic hairy root cultures (NC line) and single *STR* overexpressing lines (S lines), indicating that *G10H* plays a more important role in stimulating CPT accumulation than *STR* in *O. pumila*. Furthermore, co-overexpression of *G10H* and *STR* genes (SG Lines) caused a 56% increase on the yields of CPT compared to NC line and single gene transgenic lines, showed that simultaneous introduction of *G10H* and *STR* can produce a synergistic effect on CPT biosynthesis in *O. pumila*. The MTT assay results indicated that CPT extracted from different lines showed similar anti-tumor activity, suggesting that transgenic *O. pumila* hairy root lines could be an alternative approach to obtain CPT. To our knowledge, this is the first report on the enhancement of CPT production in *O. pumila* employing a metabolic engineering strategy.

Camptothecin (CPT), originally isolated from the bark of the Chinese happy tree *Camptotheca acuminata*^1^, is a modified terpenoidindole alkaloid (TIA) and exhibits excellent anti-tumor activity, which is due to its ability to inhibit DNA topoisomerase I^2^. Its two derivatives, irinothecan and topothecan, have been approved by the US Food and Drug Administration (FDA) in 1994 and used extensively for the treatment of metastatic colorectal cancer, ovarian cancer, cervical cancer and small cell lung cancer throughout the world^3^. Besides their anti-tumor activity, CPT derivatives have also been found to show good activity against viruses such as the human immunodeficiency virus (HIV)^4^. More derivatives of CPT are now in clinical trials, such as 9-nitrocamptothecin, 9-aminocamptothecin and rubitecan^5^, which will potentially result in growing demand for these drugs in the future. Due to the excellent pharmacological activity of CPT derivatives, the world market for these CPT analogues has increased rapidly. The combined sales of two CPT analogs (irinotecan and topotecan) in only 2008 had reached 2.2 billion US dollars and is expected to increase further^6^,^7^. At present, the annual production of CPT throughout the world is only 600 kg, while approximately 3000 kg of CPT is needed in the international market^8^. However, all the CPT derivatives which are consumed are synthesized from natural CPT, which is mainly obtained by extraction from the trees *C. acuminata* and *Nothapodytes foetida*^1^,^9^. Since the limited supply of CPT is from the above two woody plants with slow growth rates and low yields, it is an important and urgent task to develop sustainable and alternative production sources of CPT in order to resolve the worldwide scarcity of natural sources of CPT.

Due to several advantages such as rapid growth rate, unlimited branching, and genetic stability, *in vitro* hairy root induced by *Agrobacterium rhizogenes* has been considered as an alternative means to produce high-value secondary metabolites including CPT^10^,^11^. To overcome the low yield of active components, the development and application of metabolic engineering strategies provides a promising approach to increase CPT production by introducing multiple CPT biosynthetic genes into CPT-producing plant cells or tissue^12^,^13^, followed by culturing transgenic cell lines, hairy roots or regenerated plants on a large scale. Therefore, detailed understanding of the CPT biosynthesis pathway, especially for the committed steps, will be helpful to improve CPT production by genetic manipulation.

Camptothecin belongs to the family of monoterpenoidindole alkaloids (TIA), which are found in some plant species such as *Apocynaceae*, *Loganiaceae*, *Rubiaceae* and *Nyssaceae*. Although CPT is one of the most promising natural plant-derived anti-tumor drugs, its biosynthetic pathway and regulatory mechanism of production remain unclear^14^. The biosynthesis of CPT is a complicated process and involves several catalytic steps (Fig. 1). Tryptamine is synthesized via the shikimate pathway, and the secologanin part is synthesized via the MEP pathway^15^. Strictosidine is then converted to strictosamide, but the remaining details and precise intermediates between strictosamide and CPT are not completely defined^6^. All TIAs including CPT are derived from the common precursor strictosidine, which is formed via the condensation of the indoletryptamine and the monoterpenoid secologanin^7^. This important step is catalyzed by strictosidine synthase (STR), which is considered to be a key enzyme in TIA biosynthesis. The STR gene has been successfully isolated from *Rauvolfia serpentina*^16^, *C. roseus*^17^, *O. pumila*^18^ and *O. japonica*^7^. Over-expression of *STR* gene in transgenic *C. roseus* showed ten-fold higher STR activity than wild-type cultures, which exhibited a great enhancement effect on TIA biosynthesis^18^. Geraniol 10-hydroxylase (G10H), being a cytochrome P450 monooxygenase, can hydroxylate geraniol at the C-10 position to generate 10-hydroxy-geraniol, which is considered to be a committed step in the biosynthesis of secologanin and even TIAs^19^. The *G10H* gene was firstly cloned from *C. roseus*^19^ and recently from *C. acuminata*^20^. G10H has been reported to be a rate-limiting enzyme in the biosynthesis of terpenoid indole alkaloids in transgenic *C. roseus*^21^,^22^,^23^.

Although much is known about biosynthesis of TIA in *C. roseus*, little is known about regulation of CPT biosynthesis in CPT-producing plants^24^. The difficulties in establishing a stable transformation system for the CPT-producing woody plant *C. acuminata* led to few successful reports on introducing a CPT biosynthetic gene into *C. acuminata* by metabolic engineering in the past two decades^25^, although much effort was put into optimization of transformation procedures and conditions for *C. acuminata.* The findings that CPT exists in herbs such as *O. mungos*^26^ and the established hairy root culture system of *O. pumila* provided an alternative experimental model system for CPT biosynthesis and production^27^. Until now, there has been no report on the introduction of *STR* and/or *G10H* genes into any CPT-producing plants including *O. pumila*. In this present work, we investigated the effects of overexpressing *CrG10H* and *CrSTR* individually and simultaneously in hairy root cultures of *O. pumila*.

## Results

### Optimization of *O. pumila* hairy root induction procedure

**Figure 2:**
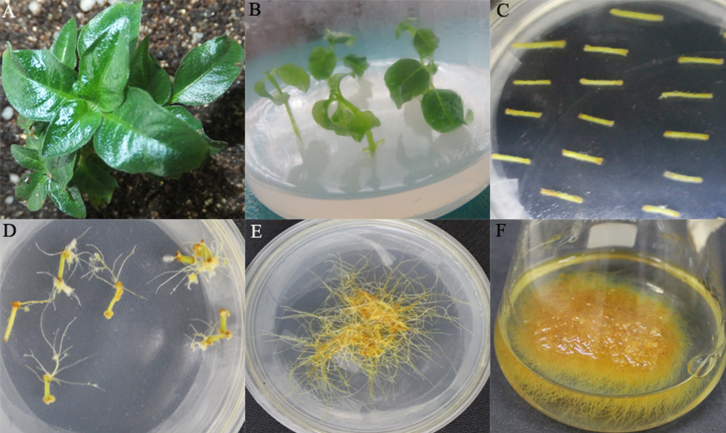
Flow chart of transgenic *O. pumila* hairy roots. (A). soil-grown *O. pumila* plant; (B). aseptic plant seedling; (C). 2d-pre-cultured *O. pumila* stems; (D). hairy roots generated from wound sites after infection by *Agrobacterium rhizogenes*; (E). hairy root on solid B5 medium; (F). hairy root cultures in the liquid B5 medium.

An efficient sterile plant culture system was established and to induce hairy root formation in *O. pumila* (Fig. 2). In this study, different explants derived from lamina, petioles and stems from *O. pumila* sterile plants were co-cultivated with *Agrobacterium* A4, 15834, and C58C1 on hormone free B5 medium for hairy root induction. Hairy root formation occurred most rapidly with strain C58C1 (induced about 10–15 days after infection, Fig. 2) among the three strains tested, suggesting that the modified strain C58C1 was more competent than the other strains as reported for *Anisodus acutangulus*^10^, and was therefore chosen for further experiments. The influence of bacterial strains on transformation frequency has been previously documented in different plant species^28^. The types of explants also affected hairy root induction frequencies. Hairy roots could be induced from wounded sites on the different explants including lamina, petioles and stems after infection with C58C1. The hairy root induction efficiency of lamina and petioles only reached to about 2% and 8%, respectively. And the explants often went brown, the hairy root derived from lamina and petioles grew slowly. The highest hairy root induction efficiency of 80.4% was achieved using modified strain C58C1 with stem explants, suggesting that stems were more susceptible than lamina and petioles. Out of three tested media (MS, GB5 and White), the hairy root in B5 medium grew much better with normal branching than the other two media (White and MS). After about 14 days, the hairy roots in B5 medium began to branch largely, whereas hairy roots in the other two media didn't branch and grew slowly, became abnormal and aged. The results suggested the liquid B5 medium was the most suitable for *O. pumila* hairy root growth. Apical tips of hairy roots (1 mg segments) were inoculated in the liquid medium, and the fresh weight of the tissue typically reached about 3 g after 45 days of culture. The above efficient hairy root induction system for the medicinal plant *O. pumila* was successfully developed and optimized for further genetic transformation.

### Acquisition of transgenic *O. pumila* hairy roots with *CrSTR* and *CrG10H*

Three plasmids containing the cDNAs encoding CrSTR and/or CrG10H under the control of the CaMV 35S promoter were separately introduced into *O. pumila* hairy roots by using disarmed *A. tumefaciens* C58C1 strain after infection of young stems of *O. pumila*. Hairy root lines generated from transformations with an empty vector that did not contain *CrSTR* or *CrG10H* genes was used as a control (NC line). After two weeks, transgenic *O. pumila* hairy roots were generated with phenotypic characteristics such as being long, thin and golden yellow (Fig. 2).The abbreviations S, G, and SG refer to the transgenic hairy root lines generated from *CrSTR* single gene, *CrG10H* single gene, and *CrSTR/CrG10H* double gene transformations, respectively. In total, 53 *STR* single gene transformed lines (S line), 34 *G10H* single gene transformed lines (G line) and 95 *STR-G10H* double gene transformed lines (SG line) were generated, and 48 S, 31 G, 88 SG hygromycin-resistant (2 mg/L) hairy root lines with normal phenotype were maintained for PCR analysis (Table 1).

**Figure 3:**
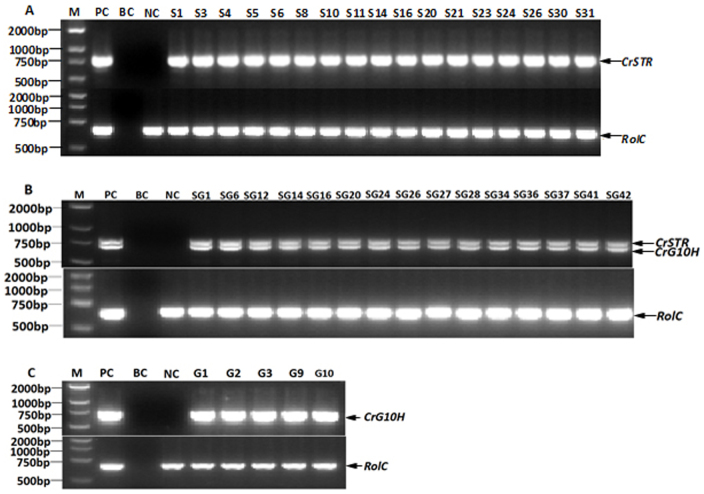
Representative results of transgenic hairy root by PCR identification. (A). PCR analysis of *CrSTR* and *RolC* for S lines; (B). PCR analysis of *CrSTR, CrG10H* and *RolC* gene for SG lines; (C). PCR analysis of *CrG10H* and *RolC* gene for G lines; M: marker DL2000; PC: C58C1 strains harboring expression vector as positive control; NC: empty control (hairy root generate from empty-vector transformation) as negative control; BC: ddH2O as blank control.

Genomic DNA of all the above independent hairy roots were isolated and used for PCR analysis using primers specially designed to overlap part of the *CrG10H*, *CrSTR* and the CaMV 35S promoter sequences. The C58C1 strains harboring plasmids *pCAMBIA1304^+^-CrSTR*, *pCAMBIA1304^+^-CrG10H* and *pCAMBIA1304^+^-CrSTR-CrG10H* were also amplified as positive controls (PC). Control hairy roots generated from empty vector transformations were used as negative controls (NC), and water was used as a blank control (BC). The *rolC* gene as a marker gene of C58C1 was also detected in all the PCR-positive clones (Fig. 3). No amplicons of *CrG10H* and *CrSTR* were detected in the NC and BC lines. In total, the PCR-positive clones amounted to 25.7% (43/167), with 35% (17/48) for S lines, 16% (5/31) for G lines, and 24% (21/88) for SG lines (SG). These results preliminarily suggest that the *CrSTR* and *CrG10H* genes were introduced into the genome of S and G lines, while both *CrSTR* and *CrG10H* genes were introduced into SG lines. Here we randomly selected 4 S, 3 G and 5 SG of PCR-positive lines with normal growth phenotypes for further establishment of hairy root culture lines for qRT-PCR and HPLC experiments (Table 1).

### Transcript analysis of *CrG10H* and *CrSTR* gene in hairy roots

**Figure 4:**
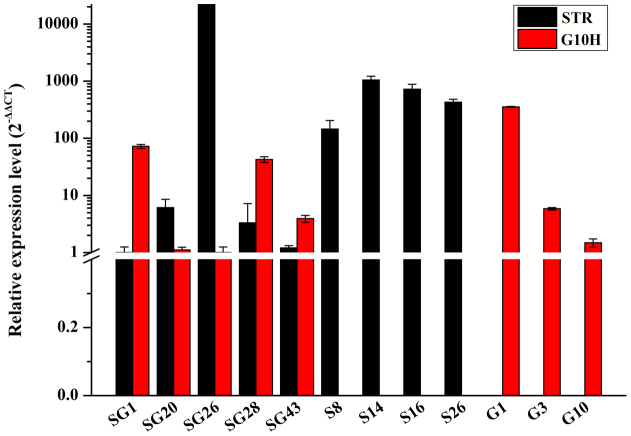
Transcription level of *CrSTR* and *CrG10H* in transgenic *O. pumila* hairy roots. SG: *CrG10H* + *CrSTR* double gene transformed lines; S:*CrSTR* single gene transformed lines; G: *CrG10H* single gene transformed lines.

qRT-PCR was determined to further analyze the expression level of the exogenous target gene (*CrG10H* and *CrSTR*) and reference gene alpha-tubulin (*Tub*) was used for the internal control gene. qRT-PCR results indicated that *CrG10H* and *CrSTR* effectively expressed with varying levels in the S, G and SG lines, respectively (Fig. 4), while no expression can be detected in the NC line as expected. Among all the transgenic lines, the G1 line had the highest expression level of *CrG10H*, while SG26 had the highest expression level of *CrSTR* (Fig. 4). These results indicated that *CrG10H* and *CrSTR* genes were expressed in corresponding transgenic lines, but with varied expression levels.

### Accumulation patterns of camptothecin

**Figure 5:**
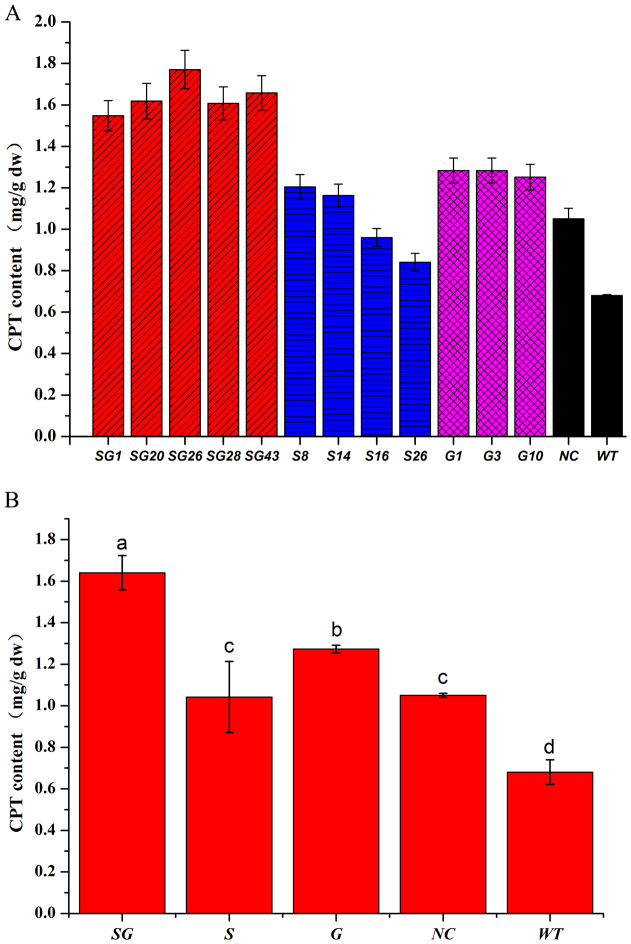
CPT contents in transgenic hairy roots of *O. pumila*. SG:*CrG10H* + *CrSTR* double gene transformed lines; S:*CrSTR* single gene transformed lines; G: *CrG10H* single gene transformed lines; NC: hairy roots induced by C58C1 with empty vector; WT: root of wild-type *O. pumila*.

The hairy roots of *O. pumila* were inoculated into 50 mL liquid B5 medium and cultured for 6 weeks followed by collection for further study. The camptothecin that accumulated in the hairy roots was detected and quantified by HPLC analysis (Fig. 5). Higher levels of CPT ranging from 1.25 to 1.28 mg/g existed in *G10H*-transformed lines than in NC hairy root (1.05 mg/g) and wild-type plant root (0.68 mg/g dw) (Fig. 5). The average CPT content (1.27 mg/g) of all the four G lines was obviously higher than NC and wild-type root, implying that overexpression of *G10H* can efficiently promote the accumulation of CPT in *O. pumila*. The levels of CPT in S lines were variable with a range of 0.83-1.20 mg/g dw, which was higher than wild-type roots but not as good as in G lines, implying that the effect of single STR is much weaker than G10H for CPT biosynthesis in *O. pumila*. Interestingly, hairy root lines co-overexpressing *G10H* and *STR* produced higher levels of CPT with variation from 1.54 to 1.77 mg/g when compared to NC hairy roots and wild-type roots, and even higher than single overexpression of *G10H* or *STR* (Fig. 5). These results indicated that co-expression of *G10H* and *STR* can produce a synergistic effect for CPT accumulation in *O. pumila.*

One-way analysis of variance (ANOVA) was used to detect the difference of the average total content of CPT among S, G, SG, NC and WT lines to analyze the functional role of STR or/and G10H in CPT biosynthesis. It showed that the average content of CPT was higher in S lines (1.04 mg/g) and NC lines (1.05 mg/g) than WT (0.68 mg/g), while much higher in G (1.27 mg/g) and SG (1.64 mg/g) lines compared with NC and WT lines (P < 0.05) (Fig. 5). Overexpression of *G10H* in G and SG lines both effectively promote CPT biosynthesis, suggesting that *G10H* is a key regulation target gene for metabolic engineering of CPT biosynthetic pathway. CPT production is higher in SG lines than G lines, implying that *STR* also plays an important role in CPT synthesis under some conditions (when *G10H* was overexpressed). Therefore, compared with a single gene, co-introduction of *G10H* and *STR* can produce a coordination effect for camptothecin biosynthesis in *O. pumila* hairy root.

### Inhibitory activity on myelogenous leukemia cells

**Figure 6:**
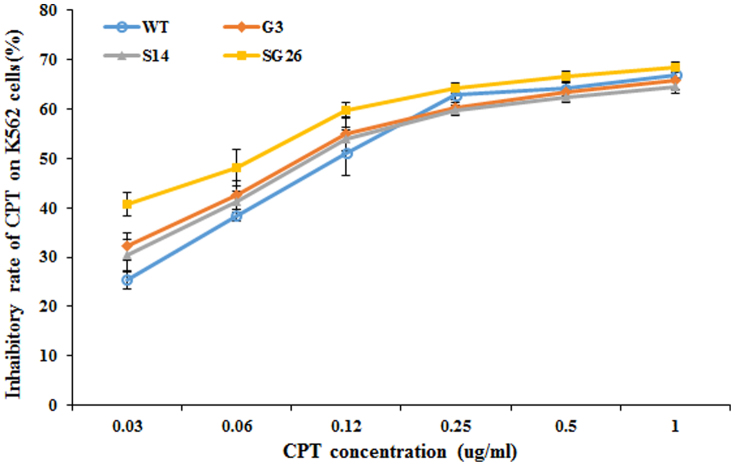
The inhibitory rate of different transgenic line on K562 cells.

The anti-tumor activity of CPT extracted from different hairy root lines was estimated by the MTT test in this study. The crude CPT extracts from some selected transgenic *O. pumila* hairy root lines and wild type *O. pumila* plant root were used for the MTT test, to compare their anti-tumor activities on the human chronic myelogenous leukemia K562 cell line. The MTT test showed that crude CPT extracts from line S14, G1, SG26 and WT at the same concentration with 6 levels showed similar anti-tumor activity (Fig. 6). When CPT from various sources was normalized at concentrations of 100 ug/ml, the inhibiting rate varied from 64.59% to 68.57%. The MTT assay results showed that there are no significant differences of anti-tumor activity among these transgenic lines and WT lines, and it can be deduced that genetic engineering did not change CPT activity.

## Discussion

Transgenic hairy root cultures have been shown to have the potential to enhance production of secondary metabolites in different plants by metabolic engineering^12^,^13^,^29^,^30^,^31^, but an effective transformation system is necessary for successful metabolic engineering of hairy root. The supply of camptothecin mainly relies on extraction from the tree *C. acuminata* at present^32^, but it is very hard to obtain transgenic lines of *C. acuminata* by genetic engineering. Weedy plants such as *O. pumila* were also found to produce CPT^26^, which provided an alternative experimental model system for CPT biosynthesis and production. Since CPT mainly existed in the roots of perennial medicinal herb *O. pumila*, which rarely distributed in limited regions in China and other Asia countries, collection of its roots as drug source may result in its extinction. *In vitro* hairy root culture is regarded as a promising strategy to obtain CPT without threatening the survival of related resource plants. A hairy root culture system of *O. pumila* with good CPT production ability has been reported for the first time by scientists in Japan, but with too long time span for induction of hairy roots (emerging 80 days after infection)^27^. In this study, we successfully optimized the procedures of hairy root induction from *O. pumila* and shortened the time taken for the emergence of hairy roots to only 10–15 days with high efficiency, which greatly simplifies the operation, and obviously improves research efficiency. Meanwhile, CPT content was obviously enhanced in transgenic *O. pumila* hairy roots using the above optimized system by means of metabolic engineering. Further MTT assay results showed that there are no obvious differences on anti-tumor activity of CPT extracted from transgenic *O. pumila* hairy root lines and WT plant roots. The above results suggested transgenic *O. pumila* hairy root lines could be an alternatively promising approach to produce more CPT in place of natural plant resources including *O. pumila*, which will reduce environmental concerns.

G10H, a cytochrome P450 monooxygenase, was considered to be a key enzyme involved in the biosynthesis of TIAs in different plant species^19^. Previous studies showed that *G10H* is a possible bottleneck for TIAs production and a good candidate target for genetic manipulation^21^. However, there is no report on the effect of overexpression of G10H in CPT-producing plants. In our study, the CPT content of G10H-transformed lines ranged from 1.25 to 1.28 mg/g, with an average CPT content (1.27 mg/g) of all the five G lines being obviously higher than NC and wild-type root (Fig 5). The above results indicate that expression of *CrG10H* obviously enhanced production of CPT in *O. pumila*, which was in good agreement with previous results that overexpression of *CrG10H* increased accumulation of TIAs in *C. roseus* plants and hairy roots^22^,^23^. Our study, for the first time, provides direct evidence that overexpression of heterologous *G10H* is sufficient to promote CPT synthesis in *O. pumila*, suggesting that *G10H* is an effective regulation target for metabolic engineering of CPT synthesis at least in *O. pumila*. Furthermore, overexpression of single *CrG10H* showed much more powerful driving effect than single *CrSTR* in increasing the production of CPT, which suggested that *G10H* may play a more important role in the regulation of CPT synthesis.

STR condenses tryptamine and the iridoid secologanin to yield strictosidine which is the universal precursor of TIAs including CPT^7^. Overexpression of *STR* in transgenic *C. roseus* showed ten-fold higher STR activity than wild-type cultures, which indicated that STR plays a critical role in biosynthesis of TIAs^18^. However, the real effect of the overexpression of the *STR* gene in CPT-producing plants remains unknown. In the present study, the camptothecin content in S lines varied from 0.83 mg/g to 1.20 mg/g, which are all higher than that of wild type root (0.68 mg/g DW). However, the CPT content was increased in two S lines (S8, S14), but decreased in the other two S lines (S16, S26) when compared to the non-transgenic control line (NC, 1.05 mg/g). The above puzzling result makes it hard to generalize the overall effect of a single STR gene on CPT biosynthesis, reflecting some kind of uncertainty in regulating metabolite flux through the biosynthetic pathway by manipulation of only a single enzyme^31^. The introduction of STR in *O. pumila* hairy roots resulted in mixed results, and the average effect of STR overexpression is not as good as G10H (Fig 5). One possible explanation for the result is that the effect of STR overexpression may be blocked by another key enzymes such as G10H.

The “push-pull” strategy has been successfully applied to increase pharmaceuticals and flavors yields in many plants such as *C. roseus, Solanum lycopersicum*, *Artemisia annua, Salvia miltiorrhiza* and *Anisodus acutangulus,* respectively^12^,^13^,^31^,^33^,^34^. But sometimes overexpression of a single gene encoding a rate-limiting enzyme to increase contents of secondary metabolites leads to unexpected consequences, and simultaneous overexpression of several enzymes within the same pathway is more effective to increases metabolites production^31^. In our study, SG lines possessed higher average CPT content (1.64 mg/g DW) than S lines (1.04 mg/g DW) and G lines (1.27 mg/g DW) (Fig 5B), and all the tested SG lines have a higher level of camptothecin than other lines, which varied from 1.54 mg/g to 1.77 mg/g. The CPT content of SG lines is relatively higher than the reported CPT content in the 5-week cultured *O. pumila* hairy roots (0.1% per dry weigh) and the intact plant (approx. 0.03–0.04% dry weight in the leaves, 0.1% dry weight in the young roots)^27^. Expression of single *CrSTR* has less effect than *CrG10H* in increasing the camptothecin content, but co-expression of *CrG10H* and *CrSTR* displayed a steady and more powerful pulling effect compared with S lines and G lines. The results suggested that multiple key enzymes can effectively promote the accumulation of camptothecin. Alternatively, introduction of a global transcription factor that positively regulates the pathway is more effective than modifying multiple biosynthetic pathway genes at one time^12^,^31^. Recently, we found a considerable increase in tanshinones in transgenic *S. miltiorrhiza* hairy roots after elicitor treatments^35^,^36^. Thus, transgenic hairy root technology coupled with elicitor treatments may be another promising strategy to increase CPT yield in the near future.

## Methods

### Plant materials

Wild-type *O. pumila* plants were collected from Fujian Province, China. Shoot tips of wild type plants were rinsed overnight with running tap water, soaked in 70% (v/v) ethanol for 30 sec and then in 0.1% (w/v) mercuric chloride for 10–15 min, and finally thoroughly rinsed four times with sterilized distilled water. The treated shoot tips were cultured on solid Murashige and Skoog (MS) medium to obtain sterile *O. pumila* plants using a previously reported method^8^. Sterile *O. pumila* plants were cultured and maintained at 25°C under a 14 h light/10 h dark photoperiod with light provided by cool white fluorescent lamps at an intensity of 350 μmol m^−2^ s^−1^.

### Construction of plant expression vectors

**Figure 7:**
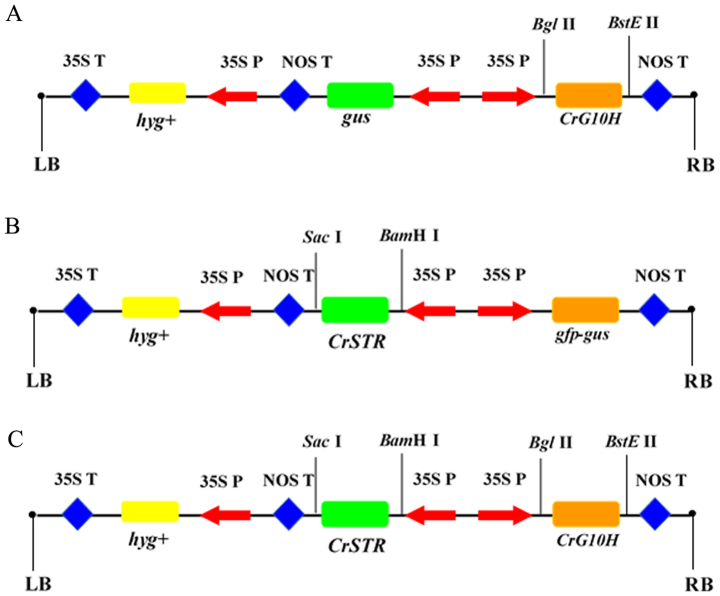
The scheme of recombinant vector. (A): pCAMBIA1304^+^-*CrSTR*, (B): pCAMBIA1304^+^-*CrG10H*, (C): pCAMBIA1304^+^-*CrSTR+CrG10H* 35SP: promoter of CaMV35S, 35ST:terminator of CaMV35S, NOST: terminator of noster, LB: left border of T-DNA, RB: right border of T-DNA, hyg+: hygromycin.

The complete *G10H* and *STR* cDNAs were cloned from the sterile seedlings of *C. roseus* according to the sequences reported in NCBI (X61932.1 for STR and AJ251269.1 for G10H). The full-length ORF of *CrG10H* cDNA was inserted into pCAMBIA1304^+^^12^,^13^,^30^ in place of the *mGFP5* and *gusA* genes to generate a *pCAMBIA1304^+^*-*CrG10H* expression vector containing the *CrG10H* gene under the digestion of *Bgl*II and *Bst*EII (Takara) (Fig. 7A). Similarly, the full-length *CrSTR* cDNA was inserted into pCAMBIA1304^+^ in place of the *gus* gene to generate a *pCAMBIA1304^+^*-*CrSTR* expression vector containing the *CrSTR* gene under the digestion of *Sac*I and *Bam*HI (Takara) (Fig. 7B). On the basis of *pCAMBIA1304^+^*-*CrSTR*, the full-length *CrG10H* cDNA was used to replace the *mGFP5* and *gusA* gene in *pCAMBIA1304^+^*-*CrSTR* under the digestion of *Bgl*II and *Bst*EII (Takara Biotech Co., Ltd) to generate the expression vector *pCAMBIA1304^+^*-*CrSTR*-*CrG10H* containing both *CrSTR* and *CrG10H* genes (Fig. 7C). The genes *CrSTR* or/and *CrG10H* were under the control of the strong cauliflower mosaic virus (CaMV) 35S promoter. The blank vector pCAMBIA1304^+^ without exogenous genes was used as the control. The disarmed *Agribacterium tumefaciens* strain C58C1 harboring both the *Agribacterium rhizogenes* Ri plasmid pRiA4^11^,^12^,^30^ and each of the four plasmids constructed above was used for plant genetic transformation.

### Plant transformation and hairy root culture

Different explants including leaf blades, petioles or stems were isolated from 4-week-old *in vitro* grown young aseptic *O. pumila* plants cultured on B5 medium supplemented with 100 mg/L LH, 0.1 mg/L NAA and 0.1 mg/L KT. The isolated explants were cut into small pieces (about 1 cm) followed by pre-incubation on B5 in the dark for 2 days, and then submerged in three *A. rhizogenes* strains (A4, 15834, and C58C1) for 10 min, blot-dried on sterile filter paper and then placed on B5 medium in the dark for 2 days. The explants were subsequently rinsed with sterile water, and transferred onto B5 medium supplemented with 300 mg/L carbenicillin. The apical tips of hairy root induced from transformed explants were excised and sub-cultured on B5 solid medium with 200 mg/L carbenicillin at 2-week intervals, the concentration of carbenicillin was gradually lowered and finally omitted after 2 month. When cultures had been cleared of agrobacterium, hairy roots were transferred onto B5 solid medium and the apical tips of rapidly growing hairy roots were inoculated into 50 mL liquid B5 media in 250 mL-shake flasks to establish hairy root lines, on a shaker with a gyratory rate of 120 rpm at 25°C for further study.

### DNA isolation and PCR analysis

Genomic DNA was isolated from harvested hairy root samples using the cetyltrimethyl ammonium bromide (CTAB) method^37^. The DNA was then used as the template in PCR analysis for detecting the presence of *CrSTR* and *CrG10H* genes in transgenic hairy roots. The sequences of 35SF23 (forward primer) located at the 35S promoter of the pBI121 vector and CrG10HR214 (reverse primer) in the interior of the CrG10H gene for the CrG10H detection were as follows: 5′-GAGGACCTAACAGAACTCGCC-3′ and 5′-ACGATTGTAGTGATCTGGCCTAATT-3′, respectively, and those of primers 35SF23 at the 35S promoter of the pCAMBIA1304 plasmid and *CrSTR* (reverse primer) in the interior of the *CrSTR* gene used for the amplification of the *CrSTR* gene were as follows: 5′-GAGGACCTAACAGAACTCGCC-3′ and 5′- TCCTCCCACACAATGGTCTTTT -3′, respectively. PCR was carried out in a total volume of 25 μL reaction mixture, containing 1 μL of each primer, 0.5 μL of 10 mmol/L dNTPs, 2.5 μL of 10× PCR buffer (Mg^2+^ plus), 1 μL genomic DNA as template and 1.25 units of Taq DNA polymerase (TaKaRa), following the protocol: the DNA template was denatured at 94°C for 10 min followed by 35 cycles of amplification (94°C for 45 s, 54°C for 60 s, 72°C for 60 s) and inoculated at 72°C for 10 min extension.

### cDNA synthesis and qPCR amplification

Total RNA was extracted from the hairy root samples using the RNA prep pure plant kit (Tiangen Biotech.) according to the manufacturer's protocol. The quality and concentration of RNA was checked and stored as described before^38^. Aliquots of total RNA (1 μg) were used as templates to generate cDNA with Oligo dT primer using avian myeloblastosis virus (AMV) reverse transcriptase (TaKaRa) in Bio-Rad T100 Thermal Cycler (Bio-Rad, USA), and then for further quantitative real-time fluorescence analysis. Gene-specific primers CrG10HQF486 (5′-GAGCGGAGAAGCGGTTGAC-3′), CrG10HQR641 (5′-TTCCCCGCCTCAACCATTA-3′), CrSTRQF171 (5′-AGGGTTCTACACTTCCGTCCAA-3′) and CrSTRQR322 (5′-TCCTCCCACACAATGGTCTTTT-3′) were used to detect the expression level of *CrG10H* and *CrSTR*, respectively. And alpha-tubulin gene (TUB-QF: CCAGATAACTTTGTTTTCGG, TUB-QR: GTGAACTCCATTTCATCCAT) was used as reference gene^39^. The qPCR amplification was performed in an Applied Biosystem Step One (Applied Biosystem). The relative Ct (threshold cycle value) method was used to estimate the initial amount of template present in the reactions by Applied Biosystems SDS 2.0.

### Determination of camptothecin by HPLC

The 6-week-old hairy roots were dried to a constant weight in an oven at 50°C, and grinded into powder and weighed. 95% ethanol (analytic grade) was then added to the powder at a ratio of 1:50 (W/V) and the mixture was concentrated to 2 mL, 95% ethanol was added to redissolve it at a ratio of 1:10 (V/V) overnight after a 60 min's ultrasonication. The extracts were clarified twice by centrifugation at 12,000 rpm for 10 min at 4°C, and the supernatant was collected and dried at 50°C using a vortex evaporator. Then the powder was dissolved in 2 mL methanol and passed through a 0.22 mm nylon filter (Pall Corporation). HPLC analysis was performed to determine the contents of CPT on a sepax sapphire C18 reversed-phase symmetry column (4.6 × 250 mm, 5 μM). The mobile phase consisted of 35% acetonitrile (HPLC grade) and 75% double distilled water. The flow rate was 1.0 mL/min, and the injection volume was 20 μL. The chromatogram was monitored at 220 nm on a Hitachi Diode Array Detector L2455. CPT standard substance was dissolved in methanol at 100 μg/ml, the retention time of the CPT was 9.58 min. The accuracy and reproducibility of HPLC analysis were confirmed by analyzing different quantities of the sample including the control group. Samples were quantified using standard curve fitting with linear regression.

### Anti-tumor activity of transgenic hairy roots by MTT analysis

The 6-week-old hairy roots from line S14, G1, SG26 and WT were dried and dissolved in methanol using the same extraction method for HPLC determination, and used to measure *in vitro* anti-tumor activity by MTT assay as described previously^40^, and methanol as negative control. The crude CPT extracts were diluted to the same concentration with a serial of grades (100 ug/ml, 50 ug/ml, 25 ug/ml, 12.5 ug/ml, 6.25 ug/ml, 3.13 ug/ml) of CPT using methanol and the final concentration of samples were adjusted using RPMI 1640 medium in a ratio of 1:100. K562 cells were cultured and the cell density was adjusted to 2 × 10^5^/mL in a total of 100 ul RPMI 1640 medium supplemented with 10% fetal bovine serum. Equal volume of mixture containing K562 cells and tested samples or methanol was added to the plate respectively and incubated at 37°C for 44 h in a humidified atmosphere consisting of 5% CO2, and 6–8 replicates were set for each sample. Then, 10 ul MTT [3-(4,5-dimethylthiazol-2-yl)-2,5-diphenyltetrazolium bromide] (Sigma-Aldrich) was added to the medium at 5 mg/ml, and the medium was incubated for an additional 4 h. And then 100 ul sodium dodecyl sulfonate (SDS) at a concentration of 10% was added to wells of culturing plates and formazan absorbance was measured with a spectrophotometer (Biomek 1000) at test and reference wavelengths of 570 nm.

### Statistical analysis

All the experiments including PCR identification, qRT-PCR, HPLC analysis and anti-tumor activity analysis were repeated three times. Results of CPT content are presented as mean values ± S.D. The error bars are due to biological triplicates. The statistical significance of CPT content difference was analyzed by one sample t test and the errors of different hairy root clones were used in the one-way analysis of variance (ANOVA) using SPSS 11.5 software (SPSS, Inc.).

## Author Contributions

G.K. and L.C. designed research, L.C., X.N., Q.J., X.T., Y.Y. and C.W. performed research, L.C., G.K. and D.S.Z. analyzed data, G.K., L.C., X.N. and D.Z. wrote the paper.

## Figures and Tables

**Figure 1:**
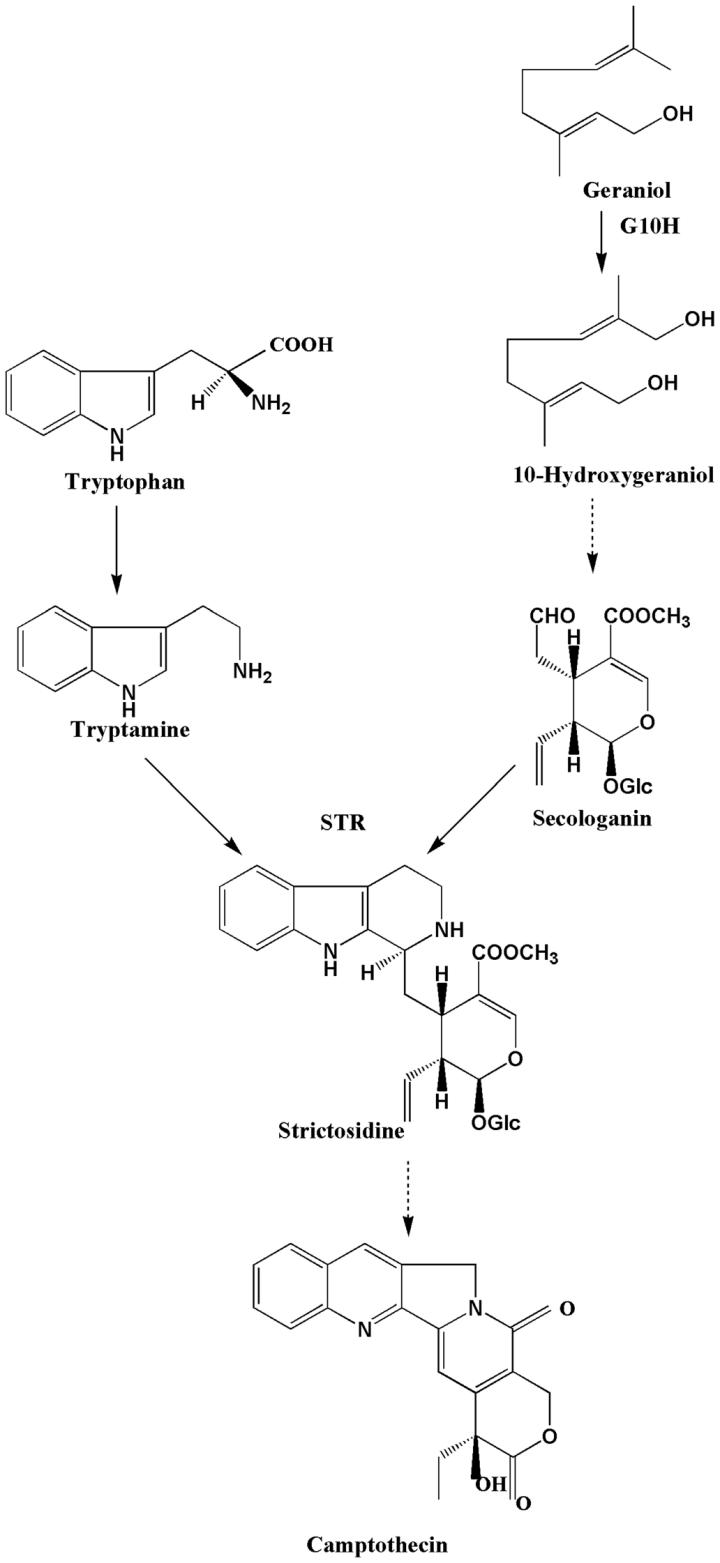
Camptothecin biosynthetic pathway in *Ophiorrhiza pumila.* Dotted line arrows indicate multiple steps between intermediates. G10H:geraniol 10-hydroxylase, STR:strictosidine synthase.

**Table 1:** Gene constructs and derived root cultures

	Number of root lines			
Gene constructs	Total	Hgr+	PCR-positive	Established root lines
*STR*	53	48	17	S8, S14, S16, S26
*G10H*	34	31	5	G1, G3, G10
*G10H* + *STR*	95	88	21	SG1, SG20, SG26, SG28, SG43
